# Association of Cortical Lesions With Regional Glutamate, GABA, *N*-Acetylaspartate, and Myoinositol Levels in Patients With Multiple Sclerosis

**DOI:** 10.1212/WNL.0000000000209543

**Published:** 2024-06-13

**Authors:** Mads A. Madsen, Michal Považan, Vanessa Wiggermann, Henrik Lundell, Morten Blinkenberg, Jeppe Romme Christensen, Finn Sellebjerg, Hartwig R. Siebner

**Affiliations:** From the Danish Research Centre for Magnetic Resonance (M.A.M., M.P., V.W., H.L., H.R.S.), Copenhagen University Hospital - Amager and Hvidovre; Department of Health Technology (H.L.), Technical University of Denmark, Kgs. Lyngby; Danish Multiple Sclerosis Center (M.B., J.R.C., F.S.), Department of Neurology, Copenhagen University Hospital - Rigshospitalet, Glostrup; Department of Neurology (H.R.S.), Copenhagen University Hospital - Bispebjerg and Frederiksberg; and Department of Clinical Medicine (F.S., H.R.S.), Faculty of Health and Medical Sciences, University of Copenhagen, Denmark.

## Abstract

**Background and Objectives:**

Cortical lesions contribute to disability in multiple sclerosis (MS), but their impact on regional neurotransmitter levels remains to be clarified. We tested the hypothesis that cortical lesions are associated with regional glutamate and gamma-aminobutyric acid (GABA) concentrations within the affected cortical region.

**Methods:**

In this cross-sectional study, we used structural 7T MRI to segment cortical lesions and 7T proton MR-spectroscopy of the bilateral sensorimotor hand areas to quantify regional GABA, glutamate, *N*-acetylaspartate, and myoinositol concentrations in patients with MS (inclusion criteria: diagnosis of relapsing-remitting [RR] or secondary progressive MS [SPMS]; age 18–80 years) and age and sex-matched healthy controls. Data were collected at a single center between August 2018 and September 2020. Linear mixed-effects models were used to test for associations between metabolite concentrations and cortical lesion volumes within the same MR-spectroscopy voxel.

**Results:**

Forty-seven patients with MS (34 RRMS, 13 SPMS; 45.1 ± 12.5 years; 31 women) and 23 healthy controls (44.4 ± 13 years, 15 women) were studied. In patients, higher regional glutamate and lower regional GABA concentrations were associated with larger cortical lesion volume within the MR-spectroscopy voxel [glutamate: 0.61 (95% CI 0.19–1.03) log(mm^3^), *p* = 0.005, GABA: −0.71 (−1.24 to −0.18) log(mm^3^), *p* = 0.01]. In addition, lower *N*-acetylaspartate levels [−0.37 (−0.67 to −0.07) log(mm^3^), *p* = 0.016] and higher myoinositol levels [0.48 (0.03–0.93) log(mm^3^), *p* = 0.037] were associated with a larger regional cortical lesion volume. Furthermore, glutamate concentrations were reduced in patients with SPMS compared with healthy participants [−0.75 (−1.3 to −0.19) mM, *p* = 0.005] and patients with RRMS [−0.55 (−1.07 to −0.02) mM, *p* = 0.04]. *N*-acetylaspartate levels were lower in both patients with RRMS [−0.81 (−1.39 to −0.24) mM, *p* = 0.003] and SPMS [−1.31 (−2.07 to −0.54) mM, *p* < 0.001] when compared with healthy controls. Creatine-normalized *N*-acetylaspartate levels were associated with performance in the 9-hole peg test of the contralateral hand [−0.004 (−0.007 to −0.002) log(s), *p* = 0.002], and reduced mean creatine-normalized glutamate was associated with increased Expanded Disability Status Scale (R = −0.39, *p* = 0.02).

**Discussion:**

Cortical lesions are associated with local increases in glutamate and a reduction in GABA concentration within the lesional or perilesional tissue. Further studies are needed to investigate the causal relationship between cortical lesions and changes in neurotransmitter concentrations.

## Introduction

Multiple sclerosis (MS) is a chronic neuroinflammatory disease of the CNS.^1^ Focal white matter demyelination results in disseminated lesions that can be visualized with standard clinical MRI examinations.^2^ However, MS is not limited to focal white matter lesions but also includes diffuse damage of radiologically normal-appearing tissue and focal lesions in gray matter.^3,4^ Cortical lesions have become an important radiologic marker of both physical and cognitive impairment in MS.^5-7^ Determining the pathophysiology of cortical lesions is, therefore, an important step toward the development of novel therapies targeting cortical involvement in MS.

Proton MR spectroscopy (^1^H-MRS) can quantify the regional concentrations of brain metabolites related to local MS pathology revealed by structural MRI. In MS, the brain metabolic profile is significantly altered.^8^ *N*-acetylaspartate (NAA) is a prominent peak in the ^1^H-MRS spectrum, which is generally believed to reflect neuronal/axonal integrity and function,^8-10^ but it should be noted that NAA is also present in myelin and oligodendrocytes. Because regional NAA levels are reduced in both normal-appearing tissue and white matter lesions,^8,11,12^ a regional reduction in NAA may not only be a sensitive indicator of neuronal damage in MS but may potentially also reflect regional demyelination. Myoinositol is primarily synthesized in astrocytes and is used as a marker of glial proliferation and inflammation.^13^ Consequently, regional myoinositol concentration is increased in white matter lesions and to a lesser extent also in normal-appearing tissue in MS.^8,14^ In addition to neural and glial metabolism, ^1^H-MRS can also be used to quantify neurotransmitter concentrations of glutamate and gamma-aminobutyric acid (GABA), the primary excitatory and inhibitory neurotransmitters of the brain, respectively. Previous studies have shown that both glutamate and GABA concentrations are changed in patients with MS compared with healthy controls and are associated with physical and cognitive disability.^15-18^ However, ^1^H-MRS of glutamate and GABA has inherent limitations at 3T because of limited spectral resolution. This results in poor sensitivity toward GABA and a lack of specificity for glutamate because of overlapping glutamate and glutamine peaks.^19^ ^1^H-MRS at ultra-high-field strength (7T) drastically improves the reliability of glutamate and GABA quantifications, by increasing signal-to-noise ratio and offering superior spectral resolution.^20^ Similar benefits of 7T MRI are seen for cortical lesion detection, which increases sensitivity up to 100%.^21,22^

In this prospective, cross-sectional study, we integrated 7T ^1^H-MRS and high-resolution structural MRI in patients with MS to quantify the regional concentration of glutamate, GABA, NAA, and myoinositol in the right and left sensorimotor hand areas (SM1-HAND). We hypothesized that regional cortical involvement, reflected by cortical lesion volume in the SM1-HAND, would change the regional balance between glutamate and GABA in the affected cortical area. In addition, we hypothesized that larger cortical lesion volume would relate to reduced regional NAA levels, indicating neuronal damage, and increased myoinositol levels because of gliosis, similar to what has been found in white matter lesions.

## Methods

### Study Participants

We prospectively recruited patients with MS from the outpatient clinic at the Danish Multiple Sclerosis Center (Copenhagen University Hospital—Rigshospitalet, Copenhagen, Denmark) between August 2018 and September 2020. Inclusion criteria were a definite diagnosis of either relapsing-remitting MS (RRMS) or secondary progressive MS (SPMS) and age between 18 and 80 years. Exclusion criteria were clinical relapses, corticosteroid therapy or changes in MS-related medication within 3 months of participation, Expanded Disability Status Scale (EDSS) score above 7, other neurologic or psychiatric disorders, and contraindications to 7T MRI. Age and sex-matched healthy volunteers were recruited through advertisements in the same period. All study participants have been described in a previous publication.^6^

### Standard Protocol Approvals, Registrations, and Patient Consents

The study was approved by the Committees on Health Research Ethics of the Capital Region of Denmark (H-17033372) and monitored by the local good clinical practice unit. All participants gave informed written consent before participation. The study complied with the Helsinki declaration of human experimentation and was preregistered at clinicaltrials.gov (ID: NCT03653585).

### Experimental Design

Data were collected over 2 experimental sessions within a 4-week period. Owing to the duration of the MRI protocol, we collected high-resolution structural 7T MRI during the first experimental session and voxel-based 7T ^1^H-MRS during the second session. No participants experienced any relapses during their participation.

### Structural MRI and ^1^H-MRS Protocol

**Figure 1:**
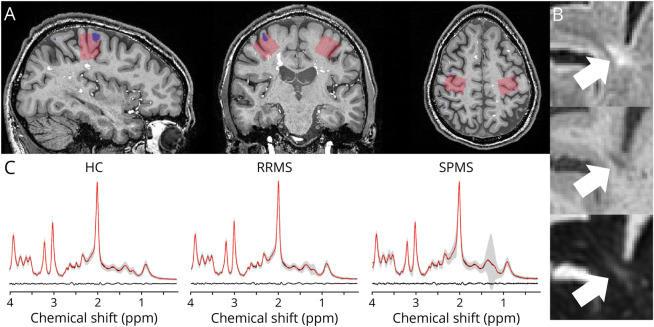
Voxel Placement and Mean Spectra (A) Example MRS voxel placement (red) in the sensorimotor hand knob, corresponding to the primary sensorimotor hand area (SM1-HAND). Segmented cortical lesions are shown in blue and white matter lesions in white. (B) Example of a leukocortical lesion located inside the MRS voxel field of view. The lesion is shown in the sagittal plane on FLAIR (top), MPRAGE (middle), and T_2_-weighted (bottom) sequences. (C) Average spectra (black) obtained with ^1^H-MRS at 7T. The gray areas represent ± SD for participant groups. Average LCModel fit results (red) and average residuals (black, bottom) are also shown. HCs = healthy controls; ppm = parts per million; RRMS = relapsing-remitting multiple sclerosis; SPMS = secondary progressive multiple sclerosis.

Structural MRI and ^1^H-MRS were performed on a 7T Achieva scanner (Philips, Best, the Netherlands) using a dual-transmit, 32-channel receive head coil (Nova Medical, Wilmington MA). The structural protocol included 3D fluid attenuated inversion recovery (FLAIR) (0.7 mm isotropic; echo time (TE): 391 ms; repetition time (TR): 5000 ms; inversion time (TI): 1.83 s), 3D magnetization prepared rapid gradient echo (MPRAGE) (0.65 mm isotropic; TE: 3.2 ms; TR: 7.5 ms), 3D T_2_-weighted turbo spin echo (0.8 mm isotropic; TE: 319 ms; TR: 3719 ms), and 3D-MP2RAGE (0.8 mm isotropic; TE: 3.5 ms; TR: 8 ms; TI: 404/3004 ms). Prospective fat-navigated motion correction^23^ was applied to all structural scans. Detailed MRI parameters can be found in our previous publication.^6 1^H-MRS measurements were acquired bilaterally from the pericentral “hand knob” region covering the SM1-HAND.^24^ A 20 × 20 × 20 mm^3^ voxel was manually placed based on a fast T_1_-weighted image (Figure 1A) with particular emphasis on being as close to the precentral gyral crown as possible without including any skull or skull fat in the voxel. ^1^H-MR spectra were acquired using the semilocalized by adiabatic selective refocusing sequence (TE: 31–33 ms, TR: 3175–4960 ms, 32 averages, 4000 Hz bandwidth, and 2048 data points).^25^ Acquisition details are given in eTable 1.

### Clinical and Behavioral Assessments

We extracted disease duration, EDSS score, and functional system subscores from clinical records if assessed within 3 months from study participation. Otherwise EDSS was performed on the second experimental session. In addition, participants were tested on the 9-hole peg test on both hands.

### Data Analysis

#### Processing of Structural MRI Data

Structural MRI data were bias field corrected, resampled to an isotropic voxel size of 0.5 mm^3^, aligned to MNI space, and coregistered as described in our previous publication.^6^ Cortical and white matter lesions were manually segmented by 3 experienced, blinded readers using FLAIR, T_2_-weighted and MPRAGE images as described in our previous publication.^6^ Cortical lesions were segmented into type I: leukocortical, type II: intracortical, and type III/IV: subpial lesions in accordance with previously suggested classifications.^26^ Whole-brain volumetric segmentations were performed with *freesurfer* (version 7.1.1) using MP2RAGE as input image. Lesion filling and manual corrections were carried out when needed.

#### Analysis of ^1^H-MRS Data

MR spectra were processed using automated in-house Matlab-based (MathWorks, Natick, MA) software and quantified using LCModel^27^ (version 6.3-1R) over the frequency range from 4.2 ppm to 0.2 ppm. The basis set was simulated in FID-A,^28^ modeling 19 metabolites and the macromolecular spectrum (eTable 1). Metabolite concentrations were estimated using the water signal as an internal reference. Tissue composition correction was applied, with white matter, gray matter, and CSF tissue fractions calculated from the *freesurfer* segmented images. Water relaxation times were adapted from the literature.^29,30^

### Statistical Analyses

Differences in demographic and clinical outcomes were compared with Kruskal-Wallis tests, Mann-Whitney *U* test, or χ^2^ tests where appropriate. Metabolite concentrations were compared between groups (HC, RRMS, SPMS) using linear mixed-effects models corrected for sex, age, and gray and white matter fraction in the ^1^H-MRS voxel. *Subject* was used as a random factor with random intercept. Post hoc corrections for multiple comparisons accounted for the 3 groups and used the “single-step” method.^31^ Differences in cortical lesion presence in the MRS voxel between patients with RRMS and SPMS were assessed with a logistic mixed-effects regression model with *subject* as a random factor.

The relationship between lesion volumes and metabolite concentrations was assessed in patients only, using linear mixed models with regional glutamate, GABA, NAA, myoinositol concentrations; patient status (RRMS or SPMS); age; and sex as covariates and subject as a random effect with random intercept. 9-HPT performance was assessed in a similar way, but also included healthy control data. To assess the relationship between nonlateralized measures (e.g., EDSS, disease duration, and whole-brain cortical lesion volume) and metabolite concentrations, we calculated the mean metabolite concentration from both hemispheres. Pearson correlations were computed and *p*-values were corrected for multiple comparisons using the “Holm” method within each variable. Lesion volumes were log(x + 1)-transformed before entering analyses.

Because the study did not include a control region, we assessed site specificity by running the same statistical analysis as described above, but in this analysis, MRS metabolite concentrations of the left hemisphere were matched to cortical lesion volume of the right hemisphere and vice versa. Fnally, we performed an exploratory analysis of the contribution of cortical lesion subtype (i.e., leukocortical, intracortical, and subpial) to our results using the same statistical method as described above.

Statistical analyses were conducted in R-studio (version 2022.07.1) using the packages *lme4*, *multcomp*, and *lmerTest.* Statistical threshold was set at *p* < 0.05 in all analyses.

### Data Availability

Pseudonymized data can only be shared with a formal data processing agreement and a formal approval by the Danish Data Protection Agency in line with the requirements of the GDPR.

## Results

### Demographic and Clinical Characteristics

**Figure 2:**
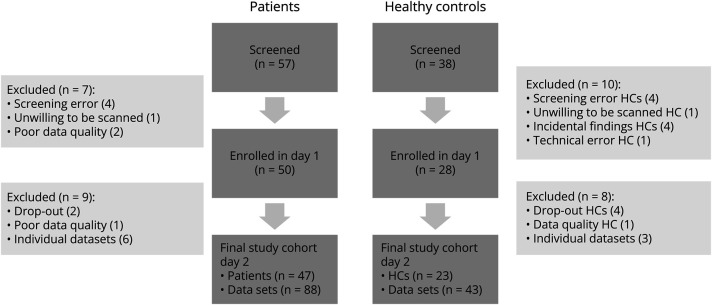
Flowchart of Study Participants and Number of Data Sets Included in the Final Analysis HC = healthy control.

A convenience sample of 57 patients and 38 healthy controls were screened for participation. Thirty-four patients with RRMS and 13 patients with SPMS (45.1 ± 12.5 years, 31 women) and 23 healthy controls (44.4 ± 13 years, 15 women) were included in the final cohort (Figure 2 and Table 1). MRS data were collected from 94 MS hemispheres and 46 healthy control hemispheres. Nine spectra were discarded because Cramér-Rao lower bounds of GABA were more than 50, leaving 88 MS spectra and 43 healthy control spectra. There were no differences in spectral quality metrics or tissue fractions in the ^1^H-MRS voxel between groups (eTable 2). In the patient group, we found a total of 55 cortical lesions in 34 of the 88 SM1-HAND voxels (39%) with a median cortical lesion volume of 9.25 mm^3^ (range, 0.87–203.37 mm^3^). Exemplar cortical lesions are shown in Figure 1A and 1B. There was no statistically significant difference in the proportion of hemispheres with a cortical lesion in the MRS voxel between patients with RRMS and SPMS (OR 3.31, 95% CI 0.54–20.25, *p* = 0.23), but patients with SPMS were older (13, 95% CI 5–20 years, *p* = 0.013) and had higher EDSS scores (2.5, 95% CI 1–3, *p* < 0.001) (Table 1).

**Table 1:** Participant Demographics and MRI Summary Statistics

	HCs (N = 23)	RRMS (N = 34)	SPMS (N = 13)	All patients (N = 47)
Age, y, mean (SD)	44.4 (13.0)	41.9 (11.9)	53.5 (9.87)^a^	45.1 (12.5)
Sex, N female (%)	15 (65.2)	23 (67.6)	8 (61.5)	31 (66.0)
EDSS score, median [min, max]		3.00 [0, 6.50]	5.00 [3.00, 6.50]^a^	3.50 [0, 6.50]
Disease duration, years median [min, max]		10.0 [0, 35.0]	17.0 [1.00, 45.0]	10.0 [0, 45.0]
Treatment, n (%)				
Alemtuzumab		1 (2.9)	0 (0)	1 (2.1)
Anti-CD20^b^		9 (26.5)	8 (61.5)	17 (36.2)
Dimethyl fumarate		1 (2.9)	0 (0)	1 (2.1)
Fingolimod		6 (17.6)	1 (7.7)	7 (14.9)
Glatiramer acetate		1 (2.9)	0 (0)	1 (2.1)
Interferon-beta		1 (2.9)	0 (0)	1 (2.1)
Natalizumab		7 (20.6)	0 (0)	7 (14.9)
Other^c^		1 (2.9)	1 (7.7)	2 (4.3)
Teriflunomide		6 (17.6)	0 (0)	6 (12.8)
Untreated		1 (2.9)	3 (23.1)	4 (8.5)
Whole-brain cortical lesion number, median [min, max]		13.0 [0, 70.0]	26.0 [4.00, 98.0]	15.0 [0, 98.0]
Whole-brain white matter lesion volume, cm^3^ median [min, max]		4.24 [0.151, 43]	8.82 [0.601, 60.7]	4.63 [0.151, 60.7]
Cortical lesion presence in the MRS voxel, n (%)				
Dominant hemisphere		11 (32.4)	7 (53.8)	18 (38.3)
Nondominant hemisphere		11 (32.4)	5 (38.5)	16 (34.0)
Cortical lesion number in the MRS voxel, median [min, max]				
Dominant hemisphere		0 [0, 2.00]	1.00 [0, 4.00]	0 [0, 4.00]
Nondominant hemisphere		0 [0, 4.00]	0 [0, 1.00]	0 [0, 4.00]
Cortical lesion volume in the MRS voxel, mm^3^ median [min, max]				
Dominant hemisphere		0 [0, 131]	8.25 [0, 58.6]	0 [0, 131]
Nondominant hemisphere		0 [0, 44.3]	0 [0, 203]	0 [0, 203]
White matter lesion volume in the MRS voxel, mm^3^ median [min, max]				
Dominant hemisphere		0 [0, 216]	32.0 [0, 2120]	0 [0, 2120]
Nondominant hemisphere		0 [0, 737]	2.88 [0, 1,190]	0 [0, 1,190]
Glutamate concentration, mM mean (SD)				
Dominant hemisphere	6.52 (0.825)	6.25 (0.898)	5.66 (0.852)	6.10 (0.915)
Nondominant hemisphere	6.51 (0.600)	6.57 (0.883)	5.77 (0.723)	6.36 (0.910)
GABA concentration, mM mean (SD)				
Dominant hemisphere	1.52 (0.491)	1.48 (0.452)	1.48 (0.450)	1.48 (0.446)
Nondominant hemisphere	1.69 (0.475)	1.97 (0.479)	1.66 (0.424)	1.89 (0.481)
NAA concentration, mM mean (SD)				
Dominant hemisphere	9.69 (1.09)	8.89 (1.12)	8.52 (1.11)	8.79 (1.12)
Nondominant hemisphere	10.1 (0.589)	9.34 (1.08)	8.68 (1.53)	9.16 (1.23)
Myoinositol concentration, mM mean (SD)				
Dominant hemisphere	4.37 (0.596)	4.40 (0.594)	4.40 (0.590)	4.40 (0.586)
Nondominant hemisphere	4.37 (0.556)	4.60 (0.653)	4.33 (1.08)	4.53 (0.781)

Abbreviations: EDSS = Expanded Disability Status Scale; GABA = gamma-aminobutyric acid; HC = healthy control; MRS = magnetic resonance spectroscopy; NAA = *N*-acetylaspartate; RRMS = relapsing-remitting multiple sclerosis; SPMS = secondary progressive multiple sclerosis.

a Significant difference between patients with RRMS and SPMS.

b Includes rituximab, ofatumumab, and ocrelizumab

c Includes cladridine and treosulfan.

### Group Differences in Metabolite Concentrations

**Figure 3:**
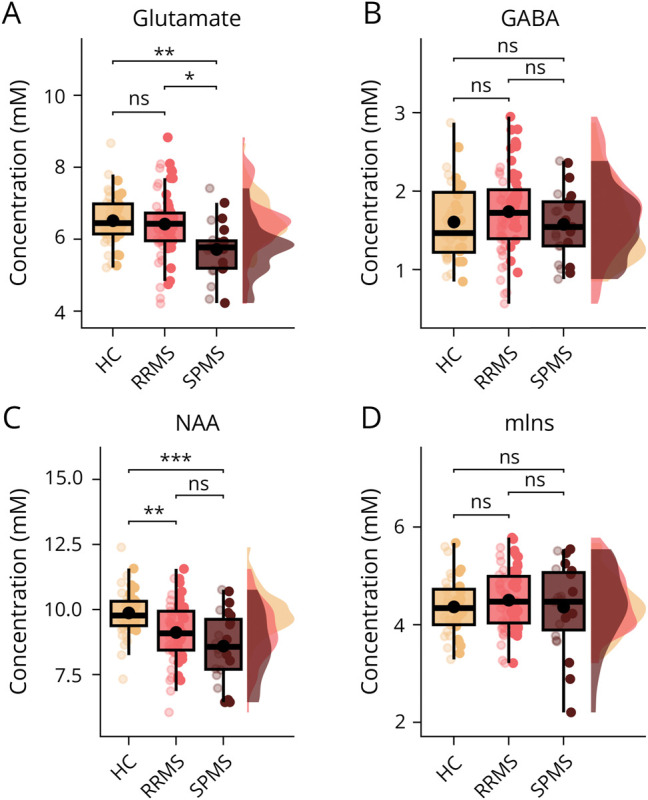
^1^H-MRS Metabolite Concentrations in SM1-HAND Box, point, and density plots of glutamate (A), GABA (B), *N*-acetylaspartate (C), and myo-inositol (D) between healthy control participants, patients with RRMS, and patients with SPMS. Box plots include median and interquartile range as horizontal lines and the mean as a black dot. Whiskers indicate the 5th and 95th percentiles. Individual datapoints are plotted over the box plot, with low opacity representing the dominant hand and high opacity the nondominant hand. HC = healthy control; mIns = myoinositol; NAA = *N*-acetylaspartate; ns = not significant; RRMS = relapsing-remitting multiple sclerosis; SPMS = secondary progressive multiple sclerosis. *p < 0.05, **p < 0.01, ***p < 0.001.

We found that the metabolic profile was different in patients with MS compared with healthy controls (*p* = 0.009, Figure 3A). Specifically, the linear mixed model showed a reduction in regional glutamate concentration in patients with SPMS relative to healthy controls (−0.75, 95% CI −1.3 to −0.19 mM, *p* = 0.005) and relative to patients with RRMS (−0.55, 95% CI −1.07 to −0.02 mM, *p* = 0.038). There were no significant differences in regional GABA concentration between groups (*p* = 0.29) (Figure 3B).

Myoinositol concentrations in SM1-HAND did not differ among groups (*p* = 0.77, Figure 3D), but NAA concentrations were lower in patients with RRMS and SPMS compared with healthy controls (*p* < 0.001, Figure 3C). The mean estimated difference was −0.81 (95% CI −1.39 to −0.24 mM, *p* = 0.003) between patients with RRMS and healthy controls and −1.31 (95% CI −2.07 to −0.54 mM, *p* < 0.001) between patients with SPMS and healthy controls.

### Alterations in Metabolite Concentrations Associated With Cortical Lesion Volume

**Figure 4:**
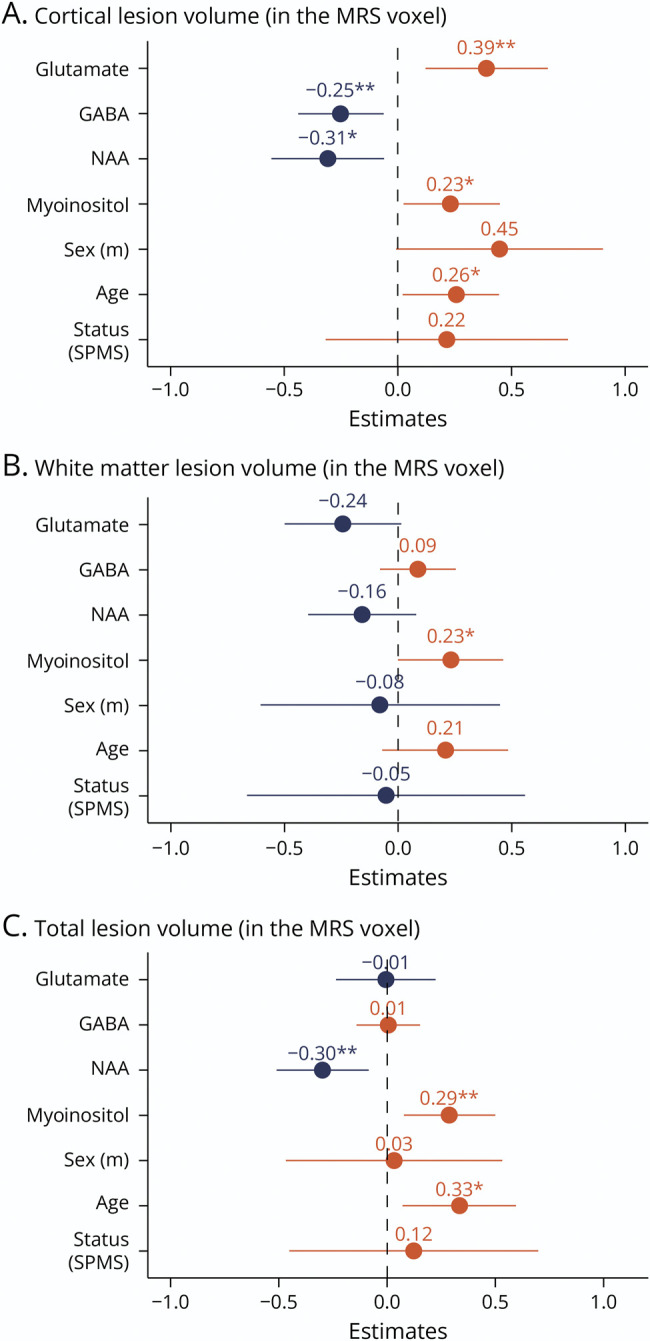
Associations Between Lesion Volume and Metabolite Concentrations in the ^1^H-MRS Voxels Covering the Right and Left SM1-HAND Standardized beta-coefficients from the mixed linear models with (A) cortical lesion volume, (B) white matter lesion volume, and (C) total lesion volume within the ^1^H-MRS voxel as the dependent variable. MRS = magnetic resonance spectroscopy; NAA = *N*-acetylaspartate; ns = not significant; SPMS = secondary progressive multiple sclerosis. *p < 0.05, **p < 0.01, ***p < 0.001.

Cortical lesion volume of the SM1-HAND was associated with an altered balance between excitatory and inhibitory neurotransmitter concentrations (Figure 4A). The higher the regional glutamate concentration, the larger the cortical lesion volume (0.61, 95% CI 0.19–1.03 log(mm^3^), *p* = 0.005). Conversely, higher GABA concentration was associated with lower cortical lesion volume (−0.71, 95% CI −1.24 to −0.18 log(mm^3^), *p* = 0.01) in the corresponding MRS voxel. Moreover, higher regional myoinositol concentration was associated with higher cortical lesion volume in SM1-HAND (0.48, 95% CI 0.03–0.93 log(mm^3^), *p* = 0.037) and higher NAA concentration with lower cortical lesion volume (−0.37, 95% CI −0.67 to −0.07 log(mm^3^), *p* = 0.016, Figure 4A). A single spectrum from a patient with SPMS contained a large lipid artifact, which did not seem to affect the spectral quality and reliability of metabolite quantification and was, therefore, included in the statistical analyses. Removing this sample from the analyses did not affect any conclusions drawn from the statistical tests.

Because the outcome variable (i.e., regional cortical lesion volume) contained many zeros, we also performed a mixed-effects Poisson regression with cortical lesion count in the MRS voxel as the outcome variable and a mixed-effects logistic regression with cortical lesion positive/negative MRS voxels as the outcome variable. These models yielded similar results for regional glutamate (Poisson regression: incidence rate ratio (IRR) 1.9, 95% CI 1.18–3.06, *p* = 0.008, logistic regression: OR 4.31, 95% CI 1.06–17.59, *p* = 0.042) and GABA concentrations (Poisson regression: IRR 0.41, 95% CI 0.19–0.87, *p* = 0.02, logistic regression: OR 0.12, 95% CI 0.02–0.68, *p* = 0.017) while the effects of regional myoinositol and NAA concentrations were no longer significant (eTables 3 and 4).

Metabolite concentrations are often referenced to total creatine concentration to adjust for variation in spectral quality and partial volume.^8^ This prompted us to perform an additional analysis exploring the relationship between cortical lesion volume and creatine-normalized metabolite concentrations. Again, we found similar results for regional glutamate (4.99, 95% CI 2.15–7.83 log(mm^3^) *p* < 0.001) and GABA concentrations (−4.14, 95% CI −7.28 to −1 log(mm^3^), *p* = 0.01). Of the structural MRS markers, only myoinositol remained statistically significant (4.18, 95% CI 1.04–7.33 log(mm^3^), *p* = 0.01, eTable 5).

We assessed site specificity by running the same statistical analysis as above, but with MRS metabolite concentrations flipped between hemispheres. The analysis showed that only myoinositol was significantly associated with cortical lesion volume of the opposite hemisphere (0.75, 95% CI 0.19–1.3 log(mm^3^), *p* = 0.01, eTable 6).

To assess whether our findings were specific to cortical lesions, we computed additional mixed linear regression models using only white matter lesion volume or the total gray and white matter lesion volume of the MRS voxel as outcome variables (Figure 4, B and C). These models showed no significant effects of regional glutamate and GABA concentrations on regional white matter or total lesion volume, indicating that the observed effects are specific to cortical lesions. By contrast, the effects of regional NAA and myoinositol on regional gray matter, white matter, or total regional lesion volume were comparable (Figure 4, B and C).

Our exploratory analysis using cortical lesion subtype volumes within the MRS voxel as an outcome variable showed that type I leukocortical lesion volume was most sensitive to differences in metabolite concentrations (glutamate: 0.55, 95% CI 0.14–0.97, *p* = 0.01, GABA: −0.44, 95% CI −0.95–0.07, *p* = 0.09, NAA: −0.3, 95% CI −0.61 to −0.01, *p* = 0.04, myoinositol: 0.57, 95% CI 0.11–1.02, *p* = 0.015). Yet, these results need to be interpreted with care because lesion volumes for these subanalyses were rather low (maximum volumes for leukocortical: 203.38 mm^3^, intracortical: 23.25 mm^3^, and subpial: 11 mm^3^).

### Associations Between Motor Dexterity and Metabolite Concentrations

**Figure 5:**
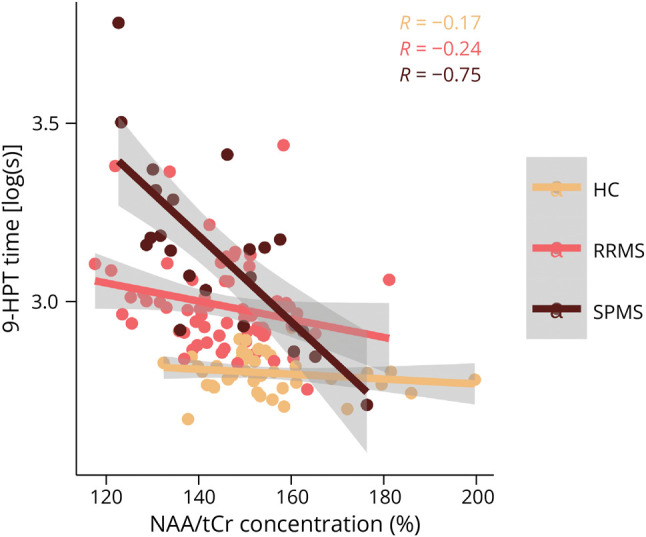
Association Between 9-HPT Performance and NAA/tCr Concentration of the Contralateral Hemisphere The plot shows the marginal effects and partial residuals from the mixed linear model fit. 9-HPT = 9-hole peg test; HC = healthy control; NAA = *n*-acetylaspartate; RRMS = relapsing-remitting multiple sclerosis; SPMS = secondary progressive multiple sclerosis; tCr = total creatine.

There were no significant associations between performance on the 9-HPT and metabolite concentrations from the mixed-effects model. However, using creatine-normalized metabolite concentrations, we found a significant negative relationship between log-transformed 9-HPT time and NAA/tCr (−0.004, 95% CI −0.007 to −0.002 log(s), *p* = 0.002). Exploring this relationship further we found a significant interaction between NAA/tCr concentration and participant status (i.e., HCs, RRMS or SPMS; *p* = 0.002), driven primarily by the SPMS group (−0.011, 95% CI −0.02 to −0.005 log(s), *p* < 0.001) (Figure 5). In patients, mean creatine-normalized glutamate concentration correlated negatively with individual EDSS scores (R = −0.39, *p* = 0.024, Table 2). There were no significant correlations between the pyramidal functional system subscore, disease duration, or whole-brain cortical lesion volume and any of the metabolites (Table 2).

**Table 2:** Correlation Coefficients and Adjusted *p*-Values Between Mean Metabolite Concentrations and Clinical and MRI Outcome Variables

	EDSS	FSp	Disease duration	Whole-brain CL volume
Mean glutamate	R = −0.36 (*p* = 0.05)	R = −0.27 (*p* = 0.28)	R = −0.10 (*p* = 0.98)	R = −0.02 (*p* = 0.874)
Mean glutamate/tCr	R = −0.39 (*p* = 0.024)^a^	R = −0.34 (*p* = 0.07)	R = −0.17 (*p* = 0.5)	R = 0.00 (*p* = 0.98)
Mean GABA	R = −0.28 (*p* = 0.13)	R = −0.23 (*p* = 0.39)	R = 0.27 (*p* = 0.27)	R = −0.22 (*p* = 0.57)
Mean GABA/tCr	R = −0.26 (*p* = 0.17)	R = −0.23 (*p* = 0.23)	R = 0.29 (*p* = 0.15)	R = −0.21 (*p* = 0.48)
Mean NAA	R = −0.21 (*p* = 0.31)	R = −0.16 (*p* = 0.58)	R = −0.19 (*p* = 0.62)	R = −0.20 (*p* = 0.57)
Mean NAA/tCr	R = −0.30 (*p* = 0.12)	R = −0.29 (*p* = 0.15)	R = −0.36 (*p* = 0.051)	R = −0.33 (*p* = 0.088)
Mean myoinositol	R = −0.06 (*p* = 0.68)	R = −0.06 (*p* = 0.69)	R = 0.03 (*p* = 0.98)	R = 0.20 (*p* = 0.57)
Mean myoinositol/tCr	R = −0.08 (*p* = 0.61)	R = −0.11 (*p* = 0.47)	R = 0.01 (*p* = 0.94)	R = 0.19 (*p* = 0.48)

Abbreviations: CL = cortical lesion; EDSS = Expanded Disability Status Scale; FSp = pyramidal functional systems; GABA = gamma-aminobutyric acid; NAA = *N*-acetylaspartate; tCr = total creatine.

^a^ Corrected *p* < 0.05.

## Discussion

Using single-voxel 7T ^1^H-MRS of the left and right SM1-HAND, we show that cortical lesion volume is associated with opposite changes in the local concentrations of glutamate and GABA within the affected cortical region. Specifically, we found that a regional increase in glutamate and a decrease in GABA concentrations were related to larger cortical lesion volume of the same region. In addition, we confirm and extend previous findings from white matter lesions by showing that cortical lesions also associate with MR-spectroscopic metabolic markers of axonal damage (i.e., reduced regional NAA concentration) and gliosis (i.e., increased regional myoinositol concentration).

Our findings suggest that local cortical lesion load shifts the regional glutamate/GABA balance toward potential excessive glutamate levels. This finding was robust, site specific, and specific to cortical lesions because including white matter lesion volume in the outcome variable diminished the effects. Histopathologic studies have shown that cortical lesions lead to a general loss of cortical neurons compared with normal-appearing gray matter.^32^ However, it is less clear whether certain neuronal populations within a cortical lesion are particularly vulnerable. Reductions in both GABAergic but not glutamatergic^33^ and glutamatergic but not GABAergic neuron densities^34^ have been reported. Similarly, reductions in synapse density specifically related to GABA or glutamate have been shown in human histopathologic samples.^35^ Cortical lesions also exhibit reduced glutamate reuptake transporters,^36^ and animal models of MS have shown altered glutamate homeostasis in active lesions and in the gray matter.^37^ The hypothesis put forward in these studies is that excessive glutamate release from activated immune cells leads to excessive extracellular glutamate levels, causing glutamate excitotoxicity that, in turn, leads to oligodendrocyte death and axonal loss.^37-39^ Accordingly, the positive association between regional glutamate concentration and increasing regional cortical lesion volume found in this study may be related to a similar mechanism rather than increased glutamate synthesis related to glutamatergic neurotransmission. This notion could also explain our seemingly counterintuitive finding that on a group level, patients with SPMS showed an overall reduction in glutamate concentration while having a numerically higher amount of whole-brain cortical lesions. Taken together, our findings show 2 coexisting glutamate-related changes that are opposite in sign. On the one hand, patients with SPMS showed a loss of glutamate potentially related to reduced glutamatergic neurotransmission. On the other hand, local inflammatory activity within cortical lesions or perilesional cortex might augment regional glutamate levels as is also seen in acute white matter lesions.^15^ The latter process may be mitigated by restoring cortical levels of GABA, which could serve as a compensatory mechanism.^40^

At 3T, 1 study has combined ^1^H-MRS with gray matter lesion measurements, within the same volume of interest.^17^ In that study, the relationship between gray mater lesion volume of the ^1^H-MRS voxel and GABA concentration was not significant. This null finding suggests that the increased sensitivity to detect cortical lesions and improved spectral quality achieved at 7T may help to improve the clinical utility of cortical lesions in neuroimaging studies on MS.^21^ Another ^1^H-MRS-MRI study at 3T found that higher magnetization transfer ratio (MTR) values within a mixed tissue ^1^H-MRS voxel of the sensorimotor cortex were associated with higher glutamate and lower GABA levels.^41^ This relationship between glutamate, GABA, and MTR is opposite in sign compared with what would have been expected from the present study. This might not only be because of the lower specificity of 3T MRI but also because MTR is a rather nonspecific microstructural marker.^42^

Previous 3T ^1^H-MRS measurements of glutamate and GABA have yielded conflicting results in MS. Elevated glutamate levels have been shown in normal-appearing white matter and acute white matter lesions,^15,43^ but reduced levels in mixed tissue voxels have also been reported.^16,18,41,44^ Similarly, regional increases^41^ and decreases^17^ in GABA levels have been found in patients with MS in mixed tissue voxels. These discrepancies may be ascribed to glutamate and GABA being difficult to reliably quantify at 3T. In addition, heterogeneity among studies in terms of acquisition and quantification strategies and clinical characteristics of the experimental cohorts may also have contributed to these discrepant findings.^8^ It is also important to note that most of these studies have not targeted the motor cortex, but primarily focused on regions within the white matter.

Only 1 study has so far investigated neurotransmitter concentrations using ultra-high-field ^1^H-MRS. In that study, glutamate and GABA levels were reduced in the frontal cortex of patients with progressive MS.^45^ In the current study, we also found an overall reduction in glutamate levels in the SM1-HAND in patients with SPMS, suggesting that regional glutamate concentration decreases after the transition from a relapsing-remitting to progressive course. This finding needs to be corroborated in longitudinal studies. By contrast, GABA levels in the present study were neither reduced in patients with a relapsing remitting nor a secondary progressive disease course, but cortical lesion volume was found to be associated with lower GABA levels in the SM1-HAND.

We found reduced NAA levels of the SM1-HAND in patients with RRMS and SPMS that were related to impairments in contralateral manual dexterity. This finding suggests that there is a structure-function relationship between MRS-derived measures of neuronal damage and behavior related to that specific area. This finding further strengthens the relevance of ^1^H-MRS measurements of NAA as a viable clinical marker of neuronal pathology related to clinical impairment. In addition, we found that cortical lesion volume of the ^1^H-MRS voxel was associated with a further reduction in NAA levels. This extends previous ^1^H-MRS studies in MS, showing reduced NAA levels in normal-appearing regions,^8^ chronic white matter lesions,^46^ and active white matter lesions,^15^ to now also include cortical lesions. In summary, these findings indicate that MS is associated with neuronal damage in the SM1-HAND, which increases with regional cortical lesion burden. This finding is supported well by previous histopathologic observations of reduced neuronal density in MS cortical lesions.^32^

We did not find any differences in myoinositol between groups, although this has been reported previously.^8^ However, myoinositol reductions are most commonly reported in white matter lesions, which is particularly evident from 7T ^1^H-MRS studies where spectra from an entire brain slab have been acquired simultaneously.^47^ Similarly, we found that both white matter and cortical lesion volumes scaled positively with regional myoinositol concentration, supporting the notion that astrogliosis also occurs in cortical lesions.^48,49^ However, the observed effects on myoinositol were not site specific and may be an epiphenomenon of increased cortical lesion burden, although we did not see any correlation with overall cortical lesion volume.

The cross-sectional study design makes it difficult to draw any causal conclusions, and larger longitudinal studies are warranted to investigate the temporal development of neurotransmitter imbalances and cortical lesions.

While a sufficient sample size is always a critical issue, especially for cross-sectional studies, the sample size in this study is one of the largest MS populations for studying cortical lesions with 7T MRI so far^21^ and is on par with other 7T MRS studies in MS that have studied metabolic abnormalities.^45,47^ The age span of the patients included in this study was relatively large (23–69 years). We did not impose strict age limits because we wished to study a representative population of patients with MS. It is unlikely that age-related changes in metabolite concentrations may have influenced our results because the HC cohort was well matched in terms of mean age and age span. We further included age as a confounder in all the mixed-effects analyses to accommodate the large age span. Metabolite concentrations were measured from a single relatively large voxel with partial volume effects, which are not necessarily comparable between our HC cohort and patients with MS, although there were no differences in tissue fractions between groups. Furthermore, although 7T MRI drastically improves cortical lesion sensitivity, it still only catches around 40%, compared with histopathology.^21^ Thus, despite using state-of-the-art methods, we cannot ascertain that some cortical lesions have not gone unnoticed or that lesion volume has been wrongly estimated.

Previous MRI studies have shown differences in tissue water molarity, T_1_ and T_2_^8^ relaxation times in lesions, and normal-appearing tissue of patients with MS relative to HCs. We believe that these potential differences between groups had a minimal impact on our results because we obtained similar spectroscopic findings with both water and creatine referencing.

Finally, we only investigated metabolite concentrations in the sensorimotor hand area, and further research is needed to determine whether this finding generalizes to other parts of the brain.

Leveraging the improved sensitivity of ultra-high-field MRI for cortical lesion detection and ^1^H-MRS, our study provides novel in vivo insight into the metabolic consequences of cortical lesions in MS. We provide evidence that cortical lesions associate with local changes in the balance between glutamate and GABA as well as changes in markers of neuronal integrity and glial proliferation. Our findings raise the possibility that glutamate excitotoxicity and reduced intracortical inhibition may be relevant pathophysiologic features of cortical lesions in MS.

## Appendix Authors

**Table:**

Name	Location	Contribution
Mads A. Madsen, PhD	Danish Research Centre for Magnetic Resonance, Copenhagen University Hospital - Amager and Hvidovre, Denmark	Drafting/revision of the manuscript for content, including medical writing for content; major role in the acquisition of data; study concept or design; analysis or interpretation of data
Michal Považan, PhD	Danish Research Centre for Magnetic Resonance, Copenhagen University Hospital - Amager and Hvidovre, Denmark	Drafting/revision of the manuscript for content, including medical writing for content; major role in the acquisition of data; analysis or interpretation of data
Vanessa Wiggermann, PhD	Danish Research Centre for Magnetic Resonance, Copenhagen University Hospital - Amager and Hvidovre, Denmark	Drafting/revision of the manuscript for content, including medical writing for content; major role in the acquisition of data; analysis or interpretation of data
Henrik Lundell, PhD	Danish Research Centre for Magnetic Resonance, Copenhagen University Hospital - Amager and Hvidovre; Department of Health Technology, Technical University of Denmark, Kgs. Lyngby, Denmark	Drafting/revision of the manuscript for content, including medical writing for content; major role in the acquisition of data; study concept or design; analysis or interpretation of data
Morten Blinkenberg, MD, PhD	Danish Multiple Sclerosis Center, Department of Neurology, Copenhagen University Hospital - Rigshospitalet, Glostrup, Denmark	Drafting/revision of the manuscript for content, including medical writing for content; major role in the acquisition of data; study concept or design; analysis or interpretation of data
Jeppe Romme Christensen, MD, PhD	Danish Multiple Sclerosis Center, Department of Neurology, Copenhagen University Hospital - Rigshospitalet, Glostrup, Denmark	Drafting/revision of the manuscript for content, including medical writing for content; major role in the acquisition of data; study concept or design; analysis or interpretation of data
Finn Sellebjerg, MD, DMsci	Danish Multiple Sclerosis Center, Department of Neurology, Copenhagen University Hospital - Rigshospitalet, Glostrup; Department of Neurology, Copenhagen University Hospital - Bispebjerg and Frederiksberg, Denmark	Drafting/revision of the manuscript for content, including medical writing for content; major role in the acquisition of data; study concept or design; analysis or interpretation of data
Hartwig R. Siebner, MD, DMsci	Danish Research Centre for Magnetic Resonance, Copenhagen University Hospital - Amager and Hvidovre; Department of Neurology, Copenhagen University Hospital - Bispebjerg and Frederiksberg; Department of Clinical Medicine, Faculty of Health and Medical Sciences, University of Copenhagen, Denmark	Drafting/revision of the manuscript for content, including medical writing for content; major role in the acquisition of data; study concept or design; analysis or interpretation of data

## Glossary

EDSS: Expanded Disability Status Scale
FLAIR: fluid attenuated inversion recovery
GABA: gamma-aminobutyric acid
IRR: incidence rate ratio
MPRAGE: magnetization prepared rapid gradient echo
MS: multiple sclerosis
NAA: *N*-acetylaspartate
RRMS: relapsing-remitting MS
SPMS: secondary progressive MS
TE: echo time
TR: repetition time
